# Endoscopic submucosal dissection for a symptomatic hypopharyngeal cavernous hemangioma: a new potential method

**DOI:** 10.1055/a-2522-0331

**Published:** 2025-02-11

**Authors:** Xu-Cheng Huo, Bao-Hui Song, Rong-Kui Luo, Yun-Shi Zhong, Ping-Hong Zhou, Xu Zhou, Ming-Yan Cai

**Affiliations:** 1 Endoscopy Center and Endoscopy Research Institute, Zhongshan Hospital, Fudan University, Shanghai, China; 2 Shanghai Endoscopic Minimally Invasive Collaborative Innovation Center, Shanghai, China; 3 Department of Pathology, Zhongshan Hospital, Fudan University, Shanghai, China; 4 Department of Otorhinolaryngology-Head and Neck Surgery, Zhongshan Hospital, Fudan University, Shanghai, China

The incidence rate of hypopharyngeal hemangioma is low, and there is no unified clinical guideline at present 1 . For smaller-sized hemangiomas, the main treatment is observation and follow-up. Intervention therapy is needed for large-sized lesions or for patients with obvious symptoms. Common treatment methods include surgery, laser therapy, and sclerotherapy 2 . Endoscopic submucosal dissection (ESD) for treatment of esophageal hemangioma has been reported, with good results 3 .

A 78-year-old man was referred to our center with a globus sensation persisting for several months. Gastroscopy revealed a submucosal lesion with a smooth bluish surface in the right piriform fossa ( Fig. 1 ). Magnetic resonance imaging revealed hypointensity on T1-weighted imaging and hyperintensity on T2-weighted imaging in the right piriform fossa, indicating a diagnosis of hemangioma ( Fig. 2 ). After multidisciplinary discussion, ESD was performed with the patient’s consent.

**Fig. 1:**
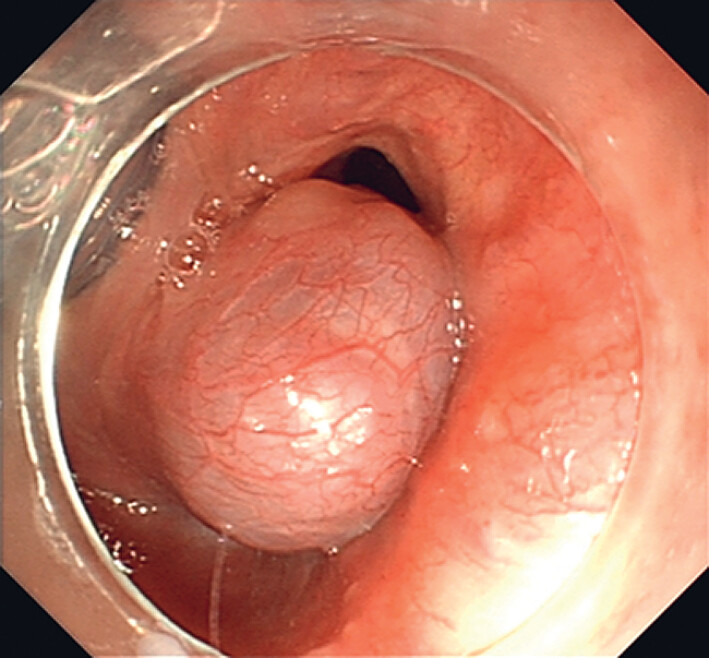
Endoscopic image of the lesion.

**Fig. 2:**
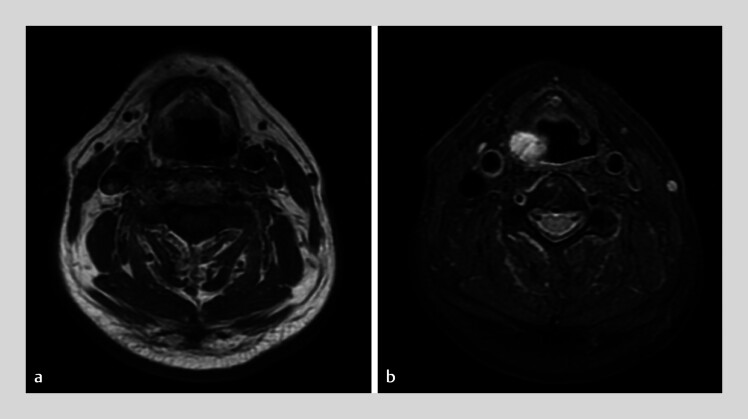
Appearance of the lesion on magnetic resonance imaging. **a** Hypointensity on the T1-weighted image. **b** Hyperintensity on the T2-weighted image.

A HybridKnife (Erbe Elektromedizin GmbH, Tübingen, Germany) was used for submucosal injection and dissection of the main lesion. An insulation-tip knife was used to cut the remaining distal section. Hemostasis was crucial owing to the highly vascular dissection plane ( Video 1 ). The pathological diagnosis confirmed cavernous hemangioma with clear margins ( Fig. 3 ). The patient was discharged on the second postoperative day. Subsequent follow-up endoscopy at 1 year revealed complete resolution of symptoms with no signs of recurrence ( Fig. 4 ).

Endoscopic submucosal dissection of a hypopharyngeal cavernous hemangioma.Video 1

**Fig. 3:**
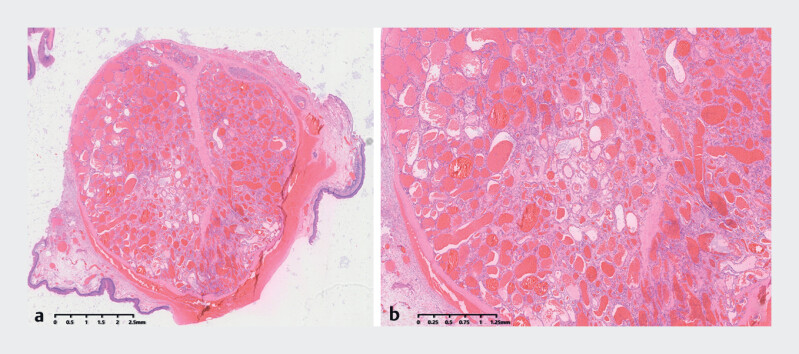
Histological examination of the resected specimen.

**Fig. 4:**
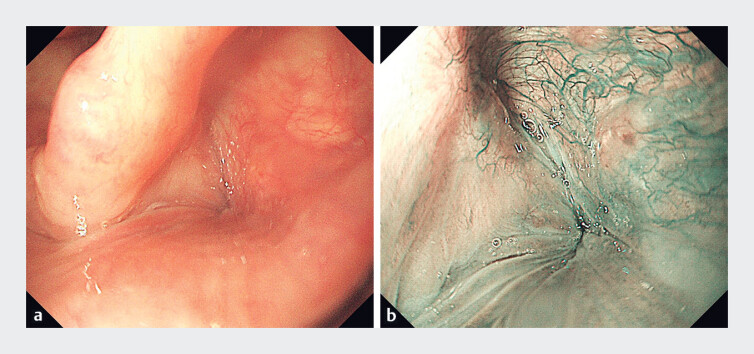
Follow-up endoscopy at 1 year. **a** White-light endoscopy. **b** Narrow-band imaging.

This is the first case of hypopharyngeal hemangioma radically removed with ESD. Our preliminary result indicates that ESD can be a potential new method for the treatment of hypopharyngeal hemangioma, with good effectiveness and safety; however, further clinical research is needed to verify our initial findings.

Endoscopy_UCTN_Code_TTT_1AO_2AG_3AD

## Notes

Endoscopy E-Videos https://eref.thieme.de/e-videos
*E-Videos* is an open access online section of the journal *Endoscopy* , reporting on interesting cases and new techniques in gastroenterological endoscopy. All papers include a high-quality video and are published with a Creative Commons CC-BY license. Endoscopy E-Videos qualify for HINARI discounts and waivers and eligibility is automatically checked during the submission process. We grant 100% waivers to articles whose corresponding authors are based in Group A countries and 50% waivers to those who are based in Group B countries as classified by Research4Life (see: https://www.research4life.org/access/eligibility/ ). This section has its own submission website at https://mc.manuscriptcentral.com/e-videos .
